# Imbricaric Acid and Perlatolic Acid: Multi-Targeting Anti-Inflammatory Depsides from *Cetrelia monachorum*

**DOI:** 10.1371/journal.pone.0076929

**Published:** 2013-10-09

**Authors:** Sarah K. Oettl, Jana Gerstmeier, Shafaat Y. Khan, Katja Wiechmann, Julia Bauer, Atanas G. Atanasov, Clemens Malainer, Ezzat M. Awad, Pavel Uhrin, Elke H. Heiss, Birgit Waltenberger, Daniel Remias, Johannes M. Breuss, Joel Boustie, Verena M. Dirsch, Hermann Stuppner, Oliver Werz, Judith M. Rollinger

**Affiliations:** 1 Institute of Pharmacy/Pharmacognosy, Center for Molecular Biosciences Innsbruck, Leopold-Franzens University of Innsbruck, Innsbruck, Austria; 2 Chair of Pharmaceutical/Medicinal Chemistry, Institute of Pharmacy, Friedrich-Schiller-University of Jena, Jena, Germany; 3 Institute of Vascular Biology and Thrombosis Research, Center for Biomolecular Medicine and Pharmacology, Medical University of Vienna, Vienna, Austria; 4 Department of Pharmaceutical Analytics, Pharmaceutical Institute, University Tuebingen, Tuebingen, Germany; 5 Department of Pharmacognosy, University of Vienna, Vienna, Austria; 6 Institute of Chemical Sciences of Rennes, Team PNSCM, University of Rennes 1, Rennes, France; USGS National Wildlife Health Center, United States of America

## Abstract

In vitro screening of 17 Alpine lichen species for their inhibitory activity against 5-lipoxygenase, microsomal prostaglandin E_2_ synthase-1 and nuclear factor kappa B revealed *Cetrelia monachorum* (Zahlbr.) W.L. Culb. & C.F. Culb. As conceivable source for novel anti-inflammatory compounds. Phytochemical investigation of the ethanolic crude extract resulted in the isolation and identification of 11 constituents, belonging to depsides and derivatives of orsellinic acid, olivetolic acid and olivetol. The two depsides imbricaric acid (**4**) and perlatolic acid (**5**) approved dual inhibitory activities on microsomal prostaglandin E_2_ synthase-1 (IC_50_ = 1.9 and 0.4 µM, resp.) and on 5-lipoxygenase tested in a cell-based assay (IC_50_ = 5.3 and 1.8 µM, resp.) and on purified enzyme (IC_50_ = 3.5 and 0.4 µM, resp.). Additionally, these two main constituents quantified in the extract with 15.22% (**4**) and 9.10% (**5**) showed significant inhibition of tumor necrosis factor alpha-induced nuclear factor kappa B activation in luciferase reporter cells with IC_50_ values of 2.0 and 7.0 µM, respectively. In a murine *in vivo* model of inflammation, **5** impaired the inflammatory, thioglycollate-induced recruitment of leukocytes to the peritoneum.

The potent inhibitory effects on the three identified targets attest **4** and **5** a pronounced multi-target anti-inflammatory profile which warrants further investigation on their pharmacokinetics and *in vivo* efficacy.

## Introduction

Lichens, wide-spread symbiotic associations between algae/cyanobacteria and fungi, occur in diverse environmental conditions, even in very inhospitable climates attended with extreme temperatures and aridity. Due to the various algal-fungal-combinations and the variable ecological and climatic growth conditions, lichens differ widely in appearance and produce a vast diversity of small chemical entities to protect the symbiosis partners against environmental influences and natural enemies. Accordingly, lichens are a rich source of bioactive secondary metabolites, such as mono- and diaromatics, terpenoids, steroids, anthraquinones, naphthoquinones, xanthones, and furans [1], with numerous reported pharmacological properties, e.g. antibiotic, antifungal, antiviral, anticancer, antioxidant, anti-inflammatory, analgesic, and antipyretic [2-6]. Such a variety of activities is also recognized from Alpine lichen species which remain an underexplored source for bioactive compounds [7].

Recently, we identified lichen constituents from the chemical group of depsides and depsidones as potent inhibitors of microsomal prostaglandin E_2_ synthase-1 (mPGES-1) using pharmacophore-based virtual screening tools [8]. Among the three isoforms of prostaglandin E_2_ synthases, mPGES-1, an inflammation induced-, membrane associated-enzyme, has been described as promising druggable target catalyzing the conversion of cyclooxygenase-derived prostaglandin (PG)H_2_ to PGE_2_, primarily affecting pathogenic processes [9].

These previous findings prompted us to further identify bioactive lichen constituents and to explore their anti-inflammatory potential. For this purpose diverse Alpine lichen species were collected and investigated to complement the previously performed structure-based approach applying an *in vitro* screening on mPGES-1. Additionally, we aimed to investigate the multi-targeting anti-inflammatory potential of lichen constituents *in vitro*, which is by far not fully investigated. Hence, the *in vitro* screening was extended to a further target within eicosanoid biosynthesis, namely 5-lipoxygenase (5-LO), since a dual inhibition of mPGES-1 and 5-LO is reported to provide safer and more effective anti-inflammatory properties [10,11]. Moreover, we also examined the lichen extracts for their inhibitory potential regarding the nuclear factor kappa B (NF-κB) pathway to broaden our insight into the bioactivity profile of lichen constituents as anti-inflammatory agents. Based on the *in vitro* screening against the focused pharmacological targets followed by an in depth phytochemical analysis of the most promising lichen extract (*C. monachorum*), we identified two depsides (**4** and **5**) contributing to the multi-target *in vitro* effect. First results from an *in vivo* pilot study underpin the anti-inflammatory efficacy of this compound class. 

## Materials and Methods

### Plant material

Lichen material of *Cetrelia monachorum* (Zahlbr.) W.L. Culb. & C.F. Culb. was collected from maple bark in Grünau/Almtal, Upper Austria (47° 44.3’ N and 13° 56.82’ E) in July 2011. The lichen material was morphologically and microchemically identified according to Obermayer and Mayrhofer [12].

Further 16 Alpine lichens were collected between August 2010 and August 2011 in Halltal and Ötztal, Tyrol, Austria, and in Vinschgau, South Tyrol, Italy, and identified according to the key in Wirth [13]. This included microscopic analyses and microchemical staining reactions using sodium hypochlorite, potassium hydroxide and *para*-phenylendiamine and enabled the unambiguous assignment of species. Voucher specimens are deposited at the Institute of Pharmacy/Pharmacognosy, University of Innsbruck, Austria. Collected lichen species, date, locality of collection, voucher numbers, amount of dried thalli and extract yield, are listed in Table S1 in Information S1.

### General experimental procedures

All reagents were of purissimum or analytical quality and purchased from Merck (Darmstadt, Germany) unless otherwise specified. 

Analysis of the metabolite profile of the ethanol crude extract of *C. monachorum* (CM) was assessed using the HPLC method: 1100 Agilent system (Agilent, Waldbronn, Germany) equipped with photodiode array detector and auto sampler; stationary phase: Phenomenex Synergi Polar-RP 80A column (4.6 × 150 mm; 4 µm particle size); mobile phase: aqueous 0.05 % formic acid (A) and MeOH (B); flow rate: 1.0 mL/min; oven temperature: 35 °C; detection wavelength: 235 nm; composition: start 10% B; 4 min 20% B; 9 min 60% B; 15 min 63% B; 30 min 63% B; 33 min 74% B; 48 min 75% B; 53 min 98% B; post time 10 min; fitted to MS-parameters: Bruker Esquire 3000^plus^ iontrap (Bruker Daltonics, Bremen, Germany); split: 1:5; ESI, alternating mode; spray voltage: 4.5 kV, 350 °C; dry gas: N_2_, 10 L/min; nebulizer: He, 40 psi; scanning range: *m/*z 100-1500.

NMR: 1D- and 2D-experiments were measured at a Bruker UltraShield 600 (Bruker Biospin, Rheinstetten, Germany) using methanol-*d*_4_ (**1**, **2**, **4**, **5**, **6**, **8**, **9**) or CDCl_3_ (**3**, **7**, **10**, **11**) as NMR-solvents (Euriso-Top, Saint-Aubin, France).

### Extraction and isolation

For *in vitro* screening of selected Alpine lichen species the air-dried thalli were ground with a ball mill (Micro-Dismembrator U, Sartorius AG) and extracted three times using EtOH 96% at room temperature and an ultrasonic bath (1 x 10 mL/1 g lichen material, 2 x 5 mL/1 g lichen material, 1 h each). 

**Figure 1:**
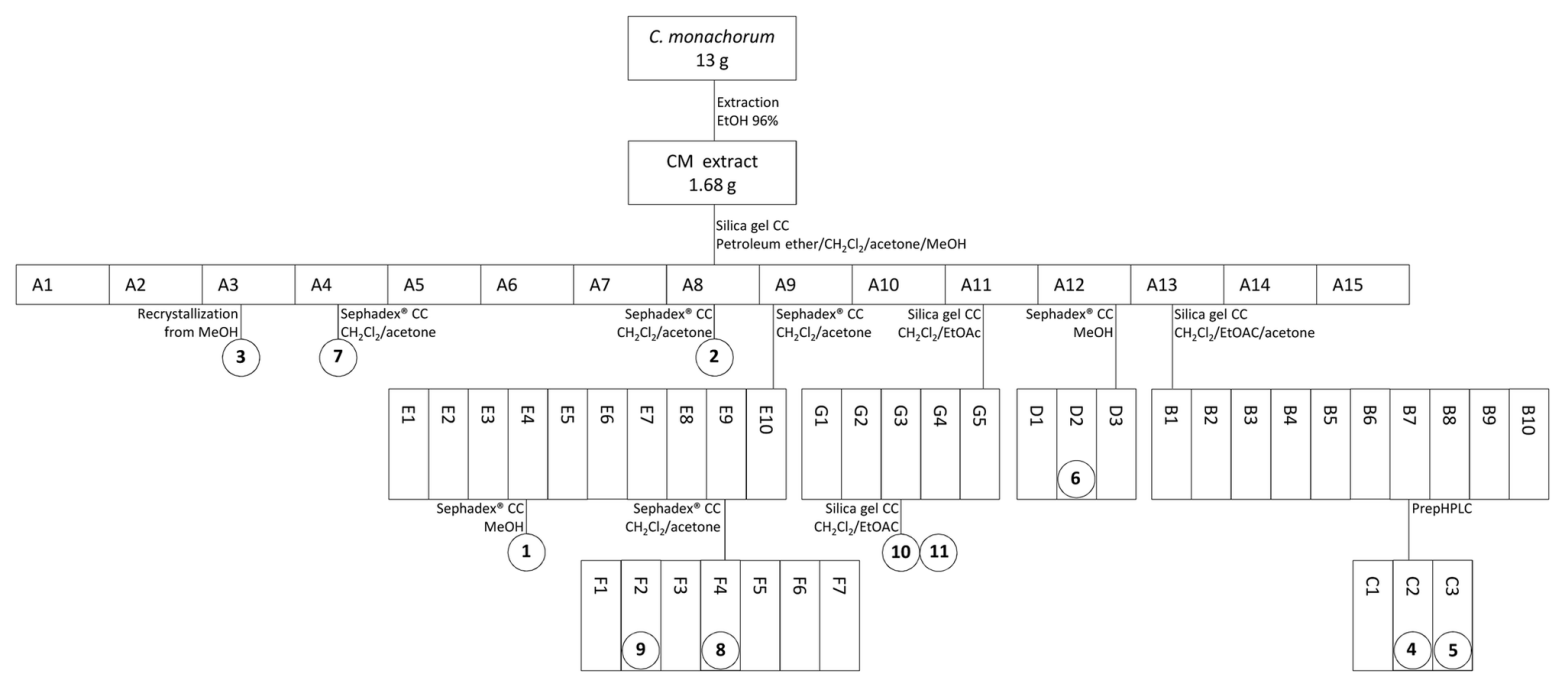
Schematic flow-chart of extraction and isolation (pure compounds encircled).

For phytochemical investigation of *C. monachorum* 13 g dried and ground thalli were extracted at room temperature with EtOH 96% using an ultrasonic bath (1 x 130 mL, 7 x 80 mL, 1 h each). Upon evaporation to dryness the crude extract (CM) yielded 1.68 g. Pure compounds **1** - **11** were isolated according to the schematic flow chart (Figure 1) using column chromatographic techniques (a detailed description is given in the online Supporting Information). 

Purity of the isolates was determined by HPLC and found to be ≥95%.

### Quantification

To determine the contents of **4** and **5** in CM, standard solutions of the isolates were diluted in appropriate concentrations. CM was dissolved in MeOH and insoluble cloudiness was filtered off. Every sample was measured three times by HPLC. Data were obtained on Shimadzu (Kyoto, Japan) UFLC-XR instrument, equipped with on-line degasser, auto sampler, column thermostat, and photodiode array detector using the HPLC protocol described above. The amounts of **4** and **5** were calculated as the percentage of dry weight of CM. Limits of quantification were determined as the signal-to-noise ratio of 10. 

### Preparation of crude mPGES-1 in microsomes of A549 cells and determination of PGE_2_ synthase activity

Preparations of human A549 cells and determination of mPGES-1 activity was performed as described previously [10]. In brief, cells were treated with 1 ng/mL interleukin-1β (IL-1β) for 48 h at 37 °C, 5% CO_2_. Cells were harvested, sonicated and the homogenate was subjected to differential centrifugation at 10,000×g for 10 min and 174,000×g for 1 h at 4 °C. The pellet (microsomal fraction) was resuspended in 1 ml homogenization buffer (0.1 M potassium phosphate buffer, pH 7.4, 1 mM phenylmethanesulfonyl fluoride, 60 µg/mL soybean trypsin inhibitor, 1 µg/mL leupeptin, 2.5 mM glutathione, and 250 mM sucrose), and the total protein concentration was determined. Microsomal membranes were diluted in potassium phosphate buffer (0.1 M, pH 7.4) containing 2.5 mM glutathione. Test compounds or vehicle (0.1% DMSO) were added, and after 15 min at 4 °C reaction (100 µl total volume) was initiated by addition of PGH_2_ at the indicated concentrations. After 1 min at 4 °C, the reaction was terminated using stop solution (100 µl; 40 mM FeCl_2_, 80 mM citric acid, and 10 µM 11β-PGE_2_ as internal standard). PGE_2_ was separated by solid-phase extraction and analyzed by RP-HPLC as described previously [10]. 

### Expression and purification of human recombinant 5-LO and determination of 5-LO activity

*E. coli* (BL21) was transformed with pT3-5-LO plasmid, and recombinant 5-LO protein was expressed at 30°C as described [14]. Cells were lysed in 50 mM triethanolamine/HCl pH 8.0, 5 mM EDTA, soybean trypsin inhibitor (60 µg/mL), 1 mM phenylmethanesulphonyl fluoride, and lysozyme (1 mg/mL), homogenized by sonication (3 × 15 sec), and centrifuged at 40,000×g for 20 min at 4°C. The 40,000×g supernatant (S40) was applied to an ATP-agarose column to partially purify 5-LO as described previously [14]. Semi-purified 5-LO was immediately used for activity assays. Thus, aliquots of the semi-purified enzyme were diluted with ice-cold PBS containing 1 mM EDTA, and 1 mM ATP was added. Samples were pre-incubated with the test compounds or vehicle (0.1% DMSO) as indicated. After 10 min at 4°C, samples were pre-warmed for 30 sec at 37°C, and 2 mM CaCl_2_ plus the indicated concentrations of arachidonic acid (AA) were added to start 5-LO product formation. The reaction was stopped after 10 min at 37°C by addition of 1 mL ice-cold methanol, and the formed metabolites were analysed by RP-HPLC as described [14]. 5-LO products include the all-trans isomers of leukotriene B_4_ (LTB_4_) and 5(S)-hydro(pero)xy-6-*trans*-8,11,14-*cis*-eicosatetraenoic acid (5-H(P)ETE).

### Determination of 5-LO products in the cell-based assay

For isolation of polymorphonuclear leukocytes (PMNL), human peripheral blood (University Hospital Jena, Germany) was withdrawn from fasted (12 h) healthy donors that had not taken any anti-inflammatory drugs during the last 10 days, by venipuncture in heparinized tubes (16 IE heparin/mL blood). The blood was centrifuged at 4,000×*g* for 20 min at 20°C for preparation of leukocyte concentrates. Leukocyte concentrates were subjected to dextran sedimentation and centrifugation on LSM 1077 cushions (PAA Laboratories, Linz, Austria). Contaminating erythrocytes of pelleted PMNL were lysed by hypotonic lysis. PMNL were washed twice in ice-cold PBS and finally resuspended in PBS pH 7.4 containing 1 mg/mL glucose and 1 mM CaCl_2_ (PGC buffer) (purity > 96-97%). 

For determination of LO products in intact PMNL (5 × 10^6^), cells were resuspended in 1 mL PGC buffer, preincubated for 15 min at 37°C with test compounds or vehicle (0.1% DMSO), and incubated for 10 min at 37°C with the indicated stimuli. Thus, the Ca^2+^-ionophore A23187 (2.5 µM) was added together with 20 µM AA and 10 min later the reaction was stopped on ice by addition of 1 mL of ice-cold methanol. Then, 30 µL 1 N HCL and 500 µL PBS, and 200 ng prostaglandin B_1_ were added and the samples were subjected to solid phase extraction on C18-columns (100 mg, UCT, Bristol, PA, USA). 5-LO products (LTB_4_ and its trans-isomers and 5-H(P)ETE) were analyzed by RP-HPLC and quantities calculated on the basis of the internal standard PGB_1_. Cysteinyl-LTsC_4_, D_4_ and E_4_ were not detected (amounts were below detection limit), and oxidation products of LTB_4_ were not determined. 

### NF-κB transactivation activity and cell viability

The transactivation of a NF-κB-driven luciferase reporter gene was quantified in HEK-293/NF-κB-luc cells (Panomics, RC0014) as previously described [15]. Cells stably transfected with a NF-κB luciferase reporter were seeded in 10 cm dishes and transfected with 5 µg plasmid encoding enhanced green fluorescent protein (pEGFP-C1; Clontech, Mountain View, CA, USA). Six hours later cells were seeded in 96-well plates and incubated at 37°C and 5% CO_2_ overnight. On the next day, the medium was exchanged with serum-free DMEM and cells were treated with the respective test compounds dissolved in DMSO. To account for potential unspecific effects of the solvent, the final concentration of DMSO was always adjusted to 0.1%. One hour after treatment the cells were stimulated with 2 ng/mL human recombinant TNF-α for 6 h, and after a lysis step the luminescence of the firefly luciferase and the fluorescence of EGFP were quantified on a GeniosPro plate reader (Tecan; Grödig, Austria). The luciferase signal derived from the NF-κB reporter was normalized by the EGFP-derived fluorescence to account for differences in cell number. 

To estimate of cell viability one part of the experiments were performed in cells that were not transfected with pEGFP-C1, but instead stained for 1 h in serum-free medium supplemented with 2 μM Cell Tracker Green CMFDA (C2925; Invitrogen) as described [16]. Since this fluorescent probe is retained inside living cells, it can be used to monitor cell membrane integrity and thus has been broadly used to quantify viable cells [17-19]. For quantification of NF-κB activity in this experimental setting the luciferase-derived signal from the NF-κB reporter was normalized to the Cell Tracker Green CMFDA-derived fluorescence to account for differences in cell number. Potential differences in cell viability were monitored by comparison of the Cell Tracker Green CMFDA fluorescence of the solvent vehicle treated control cells and cells treated with the indicated compounds.

### Mice

C57BL/6J male, 8-9 weeks old mice were housed in individually ventilated cages and fed with mouse chow and acidified water *ad libitum* in the mouse facility of the Institute of Vascular Biology and Thrombosis Research (Vienna, Austria). Animal care and all experimental procedures were approved by the Animal Experimental Committee of the Medical University of Vienna and by the Austrian Ministry of Science (license no. BMWF-66.009/0117-II/3b/2012).

### Thioglycollate-induced peritonitis

The mice of the treatment groups – sterile thioglycollate and perlatolic acid or thioglycollate alone - (3 animals per group) were pre-treated i.p. with 1 mL of 6 µM perlatolic acid or 1 µL of DMSO dissolved in 1 mL of saline. Thirty minutes later the animals were injected i.p. with another 3 mL of saline with or without sterile thioglycollate (4%), and containing again 2 μL DMSO or perlatolic acid. Five hours after the second injection mice were sacrificed by carbon dioxide exposure and peritoneal cavities were washed three times with 2 mL of cold PBS containing 3 mM EDTA and collected as intra-peritoneal lavage. The leukocyte count in the peritoneum of untreated control mice was assessed by lavage. Mice without thioglycollate treatment were used as controls. The volume of the collected lavage was measured and the cell count determined by haemocytometer.

### Statistical analysis

Data are expressed as mean ± S.E.. IC_50_ values were calculated from averaged measurements at 3-5 different concentrations of the compounds by nonlinear regression using GraphPad Prism software (San Diego, CA) one site binding competition. To calculate the IC_50_ values regarding NF-κB inhibition at least three different concentrations measured in quadruplicate in three independent transfection experiments were used utilizing nonlinear regression with Data Analysis Toolbox software (MDL Information System Inc., Nashville, TN, USA). Statistical evaluation of the data was performed by one-way ANOVA followed by a Bonferroni or Tukey-Kramer post-hoc test for multiple comparisons respectively. A p value < 0.05 was considered statistically significant. 

## Results

**Figure 2:**
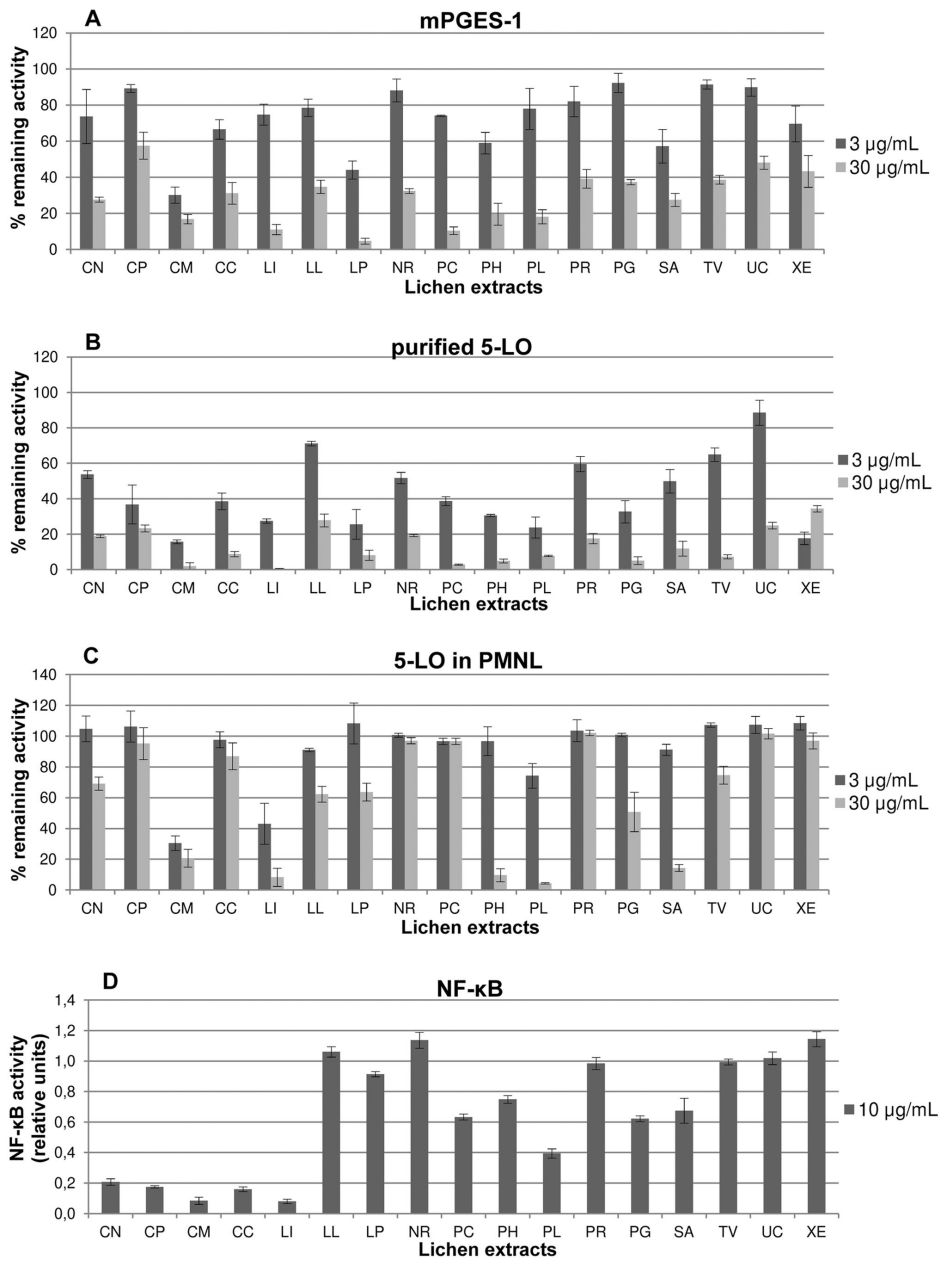
Effect of lichen crude extracts on mPGES-1 (A), purified 5-LO enzyme (B), 5-LO in PMNL (C) and NF-κB (D); n=3-5.

Several Alpine lichen species were collected in Tyrol, South Tyrol and Upper Austria. Seventeen species could be identified unambiguously using morphological and microchemical methods: *Cetraria nivalis* (L.) Ach., *Cetraria pinastri* (Scop.) Gray, *Cetrelia monachorum* (Zahlbr.) W.L. Culb. & C.F. Culb., *Cladonia carneola* (Fr.) Fr., *Lepraria incana* (L.) Ach., *Lobaria linita* (Ach.) Rabenh., *Lobaria pulmonaria* (L.) Hoffm., *Nephroma resupinatum* (L.) Ach., *Parmelia caperata* (L.) Ach., *Parmeliopsis hyperopta* (Ach.) Arnold, *Peltigera leucophlebia* (Nyl.) Gyeln., *Peltigera rufescens* (Weiss) Humb., *Platismatia glauca* (L.) W.L. Culb. & C.F. Culb., *Stereocaulon alpinum* Laurer, *Thamnolia vermicularis* (Sw.) Ach. ex Schaer., *Umbilicaria cyindrica* (L.) Delise ex Duby, and *Xanthoria elegans* (Link) Th. Fr. The ethanolic crude extracts of these species (abbreviated with first letters of genus and epithet; CN, CP, CM, CC, LI, LL, LP, NR, PC, PH, PL, PR, PG, SA, TV, UC, XE, respectively) were tested for their potency to inhibit two targets within eicosanoid biosynthesis, namely microsomal prostaglandin E_2_ synthase-1 (mPGES-1) and 5-lipoxygenase (5-LO), as well as NF-κB transactivation activity (Figure 2A - D).

For all extracts, the inhibition of mPGES-1 was assessed at concentrations of 3 and 30 µg/mL in a well-recognized cell-free assay based on the enzymatic conversion of the substrate PGH_2_ to PGE_2_ using the microsomal fraction of IL-1β-stimulated A549 lung epithelial adenocarcinoma cells as enzyme source [10]. The inhibition of 5-LO product formation was analyzed in well-established cell-free and cell-based assays [20] using puriﬁed human recombinant 5-LO and in intact human PMNL, respectively, at 3 and 30 µg/mL. While the cell-free assays allow analyzing a direct interaction with the target enzymes at the molecular level, the cell-based test system considers also regulatory aspects of 5-LO [20]. Furthermore, we screened the lichen extracts at 10 µg/mL for their potential to inhibit NF-κB transactivation activity in TNF-α-stimulated HEK-293 cells stably transfected with a NF-κB luciferase reporter gene (HEK293/NF-κB-luc cells). 

The ethanolic extracts of both *Cetrelia monachorum* (CM) and *Lepraria incana* (LI) showed outstanding pharmacological *in vitro* profiles in the applied assays (Figure 2A - D). For in depth chemical investigations we finally selected CM, because of the problematic small amount of biomass available from the powdery thallus of *L. incana*, a lichen species of the Stereocaulaceae family, in contrast to the foliose character of the thallus from *C. monachorum* , which belongs to the Parmeliaceae family.

In fact, CM concentration-dependently inhibited PGE_2_ generation with a remarkably low IC_50_ of 2.0 µg/mL. In the two 5-LO assays (cell-free and cell-based), IC_50_ values of 1.4 µg/mL and 1.3 µg/mL, respectively, were determined for CM. In TNF-α-stimulated HEK-293 cells CM inhibited the NF-κB transactivation activity with an IC_50_ of 2.6 µg/mL thereby only moderately affecting the viability of this cell line (measured between 0.3 and 10 µg/mL; viability ≥ 75.05 ± 0.08 % at 10 µg/mL). 

The metabolite profile of CM was determined via HPLC-DAD-ESI-MS analysis. Different chromatographic separation steps succeeded in the isolation of three depsides (**3 - 5**) and eight monoaromatic derivatives. By interpretation of mass spectrometric and 1D and 2D NMR data the structures were identified as ethyl haematommate (**1**), methylbetaorcinolcarboxylate (**2**), atranorin (**3**), imbricaric acid (**4**), perlatolic acid (**5**), 4-*O*-metylolivetolcarboxylic acid (**6**), ethyl-4-*O*-methylolivetolcarboxylate (**7**), ethyl olivetolate (**8**), olivetolmonomethylether (**9**), olivetol (**10**) and divarinol (**11**). Their physical and spectroscopic properties are in accordance with those reported previously ([21] and literature cited therein). Except for **3** - **5**, we identified the isolated compounds for the first time from this lichen species. 

**Figure 3:**
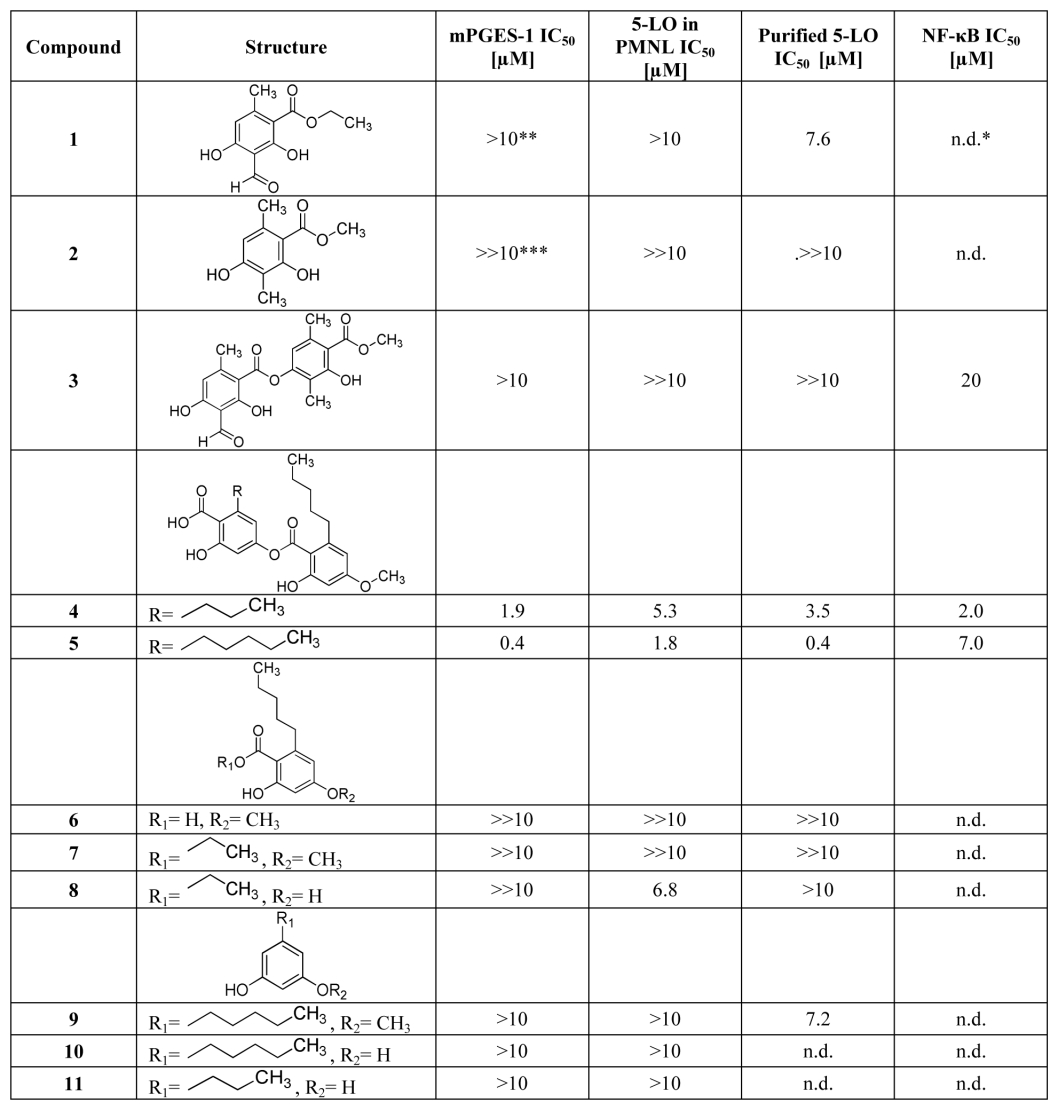
IC_50_ values of the isolated lichen compounds (1 - 11) in mPGES-1, 5-LO and NF-κB assays (n=3). * n.d., not determined; ** >10, significant inhibition at 10 µM; *** >>10, no significant inhibition at 10 µM.

**Figure 4:**
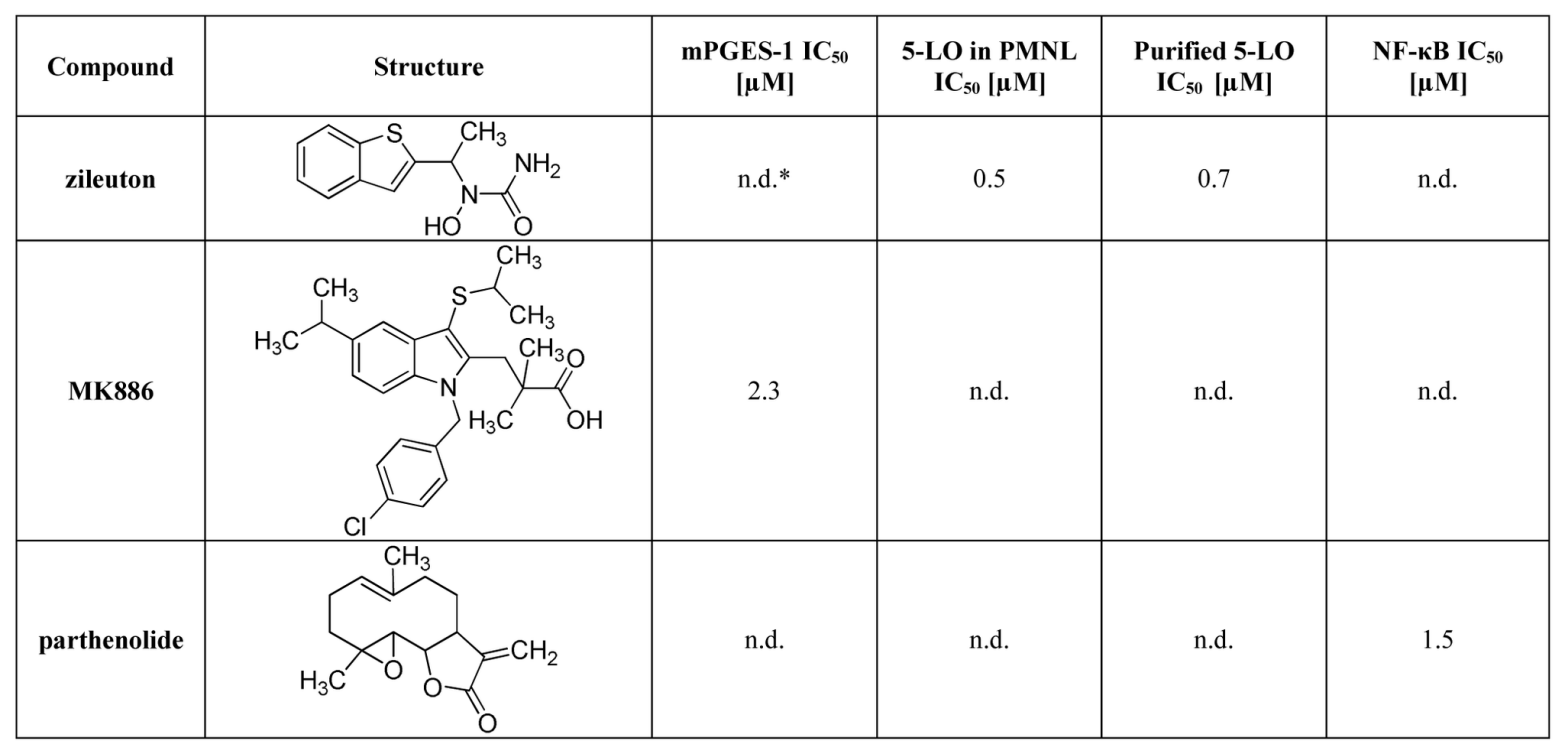
IC_50_ values of positive controls in mPGES-1, 5-LO and NF-κB assays (n=3). * n.d., not determined.

The isolates of *C. monachorum* were investigated for their potential to inhibit the activities of mPGES-1 and 5-LO. We previously identified perlatolic acid (**5**) as a major mPGES-1 inhibitor by using a pharmacophore based virtual screening (Bauer et al., 2012). Along these lines, we could identify this compound as a major mPGES-1-inhibiting ingredient (IC_50_ = 0.4 µM) in CM. Additionally, two other depsides were analyzed, namely atranorin (**3**) and imbricaric acid (**4**). As reported before (Bauer et al., 2012), **3** did not affect the mPGES-1 activity, whereas **4** exhibited a pronounced and concentration-dependent inhibition in our experiments (IC_50_ = 1.9 µM, Figure 3), comparable to the synthetic mPGES-1 reference inhibitor MK886 (IC_50_ = 2.3 µM, Figure 4) [22]. The monoaromatic compounds (**1**, **2**, and **6** - **11**), however, did not suppress enzyme activity up to a concentration of 10 µM. 

Analysis of 5-LO inhibition by the 11 lichen compounds revealed a significant inhibitory activity for the two depsides **4** and **5** in the cell-free assay with remarkable IC_50_ values of 3.5 µM and 0.4 µM, respectively. For comparison, the reference drug zileuton (the only approved 5-LO inhibitor on the market) revealed an IC_50_ = 0.7 µM (Figure 4), which is in agreement with data from the literature (0.5 - 1 µM, [23]). Thus **4** and **5** act as potent dual inhibitors of mPGES-1 and 5-LO, which implies a favorable pharmacological profile [11]. In the cell-based assay, 5-LO inhibition could be confirmed with a slightly lower potency (IC_50_ = 5.3 µM and 1.8 µM, respectively). In parallel to the results from the mPGES-1 evaluation, depside **3** and the monoaromatic constituents (**1**, **2**, and **6** - **11**) did not affect 5-LO activity up to 10 µM.

The three depsides (**3** - **5**) were additionally tested for their potential to suppress NF-κB activation in TNF-α-stimulated HEK-293/NF-κB-luc cells. In contrast to atranorin (**3**), imbricaric acid (**4**) and perlatolic acid (**5**) showed pronounced and concentration-dependent inhibition with IC_50_ values of 2.0 µM and 7.0 µM, respectively (Figure 3). These activities presented in Figure 3 are calculated after normalization of the NF-κB activity to the fluorescence derived from the internal control EGFP or from the cell viability dye Cell Tracker Green CMFDA, which were used to assure that the observed effects are not artifacts due to different cell number or differences in cell viability, respectively.

Luciferase reporter gene expression reflects a downstream event of the NF-κB signaling cascade and cannot provide insights into the molecular targets contributing to the observed NF-κB inhibition. Thus, we tested the active compounds **4** and **5** for their potential to suppress the activity of the inhibitor of NF-κB kinase subunit beta (IKK2) as previously described [24], since it is a prominent upstream target of the NF-κB signaling cascade. However, the compounds did not inhibit IKK2 at a concentration of up to 50 μM (data not shown). Accordingly, this kinase is not a direct target of the NF-κB inhibiting depsides **4** and **5**. 

**Figure 5:**
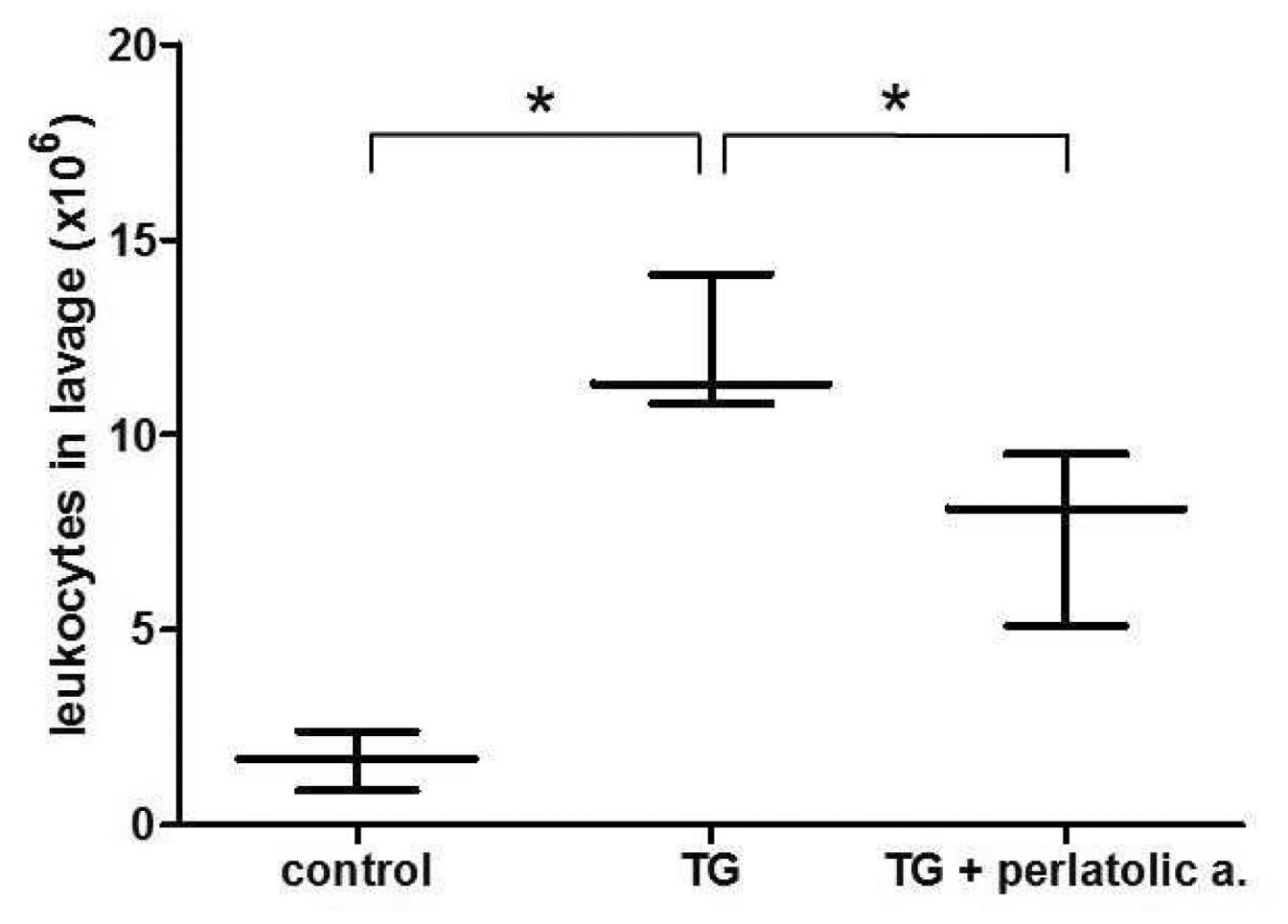
Perlatolic acid (**5**) inhibits leukocyte recruitment in thioglycollate-induced peritonitis mouse model. Box and whisker plots represent median, lowest and highest detected values (n=3; **p<0.001, *p<0.05; ANOVA/Tukey).

Because **5** appeared the most potent anti-inflammatory candidate based on the *in vitro* analysis, we aimed to assess the inhibitory effect of **5** on inflammation *in vivo*. For this purpose, we employed the thioglycollate (TG)-induced murine peritonitis model [25]. Five hours after TG injection, we found a more than fivefold increase in the total leukocyte count in the peritoneal lavage fluid in the thioglycollate-treated animals. In perlatolic acid (**5**) co-administered animals this inflammatory response was significantly reduced by 30-40% (p< 0.05, Figure 5). Brandao and coworkers recently attested perlatolic acid (**5**) a moderate selective cytotoxicity on UACC-62 and B16-F10 melanoma cells [26]. In the murine *in vivo* model of acute inflammation performed in this study no hints of toxic effects such as signs of serious discomfort or distress, change in animal behaviour or mobility, increased breathing, elevated temperature or nasal discharge were observed.

Since **4** and **5** were disclosed as those constituents prominently contributing to the observed multi-potent effect of CM, their contents were quantified by HPLC-DAD analysis in the extract and determined to be 15.22% and 9.10%, respectively. Considering the contents of **4** and **5**, and correlating their inhibitory effects with the measured bioactivities of the parent extract, the mPGES-1 inhibitory effectiveness of **4** and **5** are in full agreement with the original extract, assuming these depsides to be the major ingredients contributing to the suppression of PGE_2_ formation. Recalculations for the inhibitory activities on 5-LO and NF-κB attested CM more pronounced activities than can be achieved simply by considering the bioactivities of **4** and **5**, which leads to the assumption of further minor constituents enhancing the inhibitory effects or a synergistic action between the bioactive depsides.

## Discussion

In this study, we identified *C. monachorum* as an Alpine lichen species with pronounced inhibitory activity against three major pro-inflammatory targets, namely mPGES-1, 5-LO and NF-κB. To our best knowledge, no other plant- or natural product-derived extract has been reported with such strong mPGES-1-inhibitory activity, and also among 5-LO-suppressing plant extracts [27], CM belongs to the most potent candidates. Phytochemical characterization of the secondary metabolite profile of CM and isolation of its constituents allowed the identification of the main active ingredients of this lichen extract, namely imbricaric acid (**4**) and perlatolic acid (**5**), which both potently interfere with all three targets.

Based on these properties, *C. monachorum* represents a valuable source for novel bioactive ingredients with high potential for pharmacological intervention with inflammatory disorders.

Efficient inhibition of mPGES-1 by the isolated depsides (**3** - **5**) is in line with the SARs, which we previously established by means of structural insights from pharmacophore-based ligand-target interactions [8]. In that study, structural investigations disclosed functional interaction sites of mPGES-1 crucial for the inhibitory properties of ligands that approved *n*-pentyl side chains of depsides as important feature for interference. Underpinning this hypothesis, we determined in the present study a slightly weaker effect for the newly identified imbricaric acid (**4**) which is characterized by a three carbon chain substitution at C6’ in contrast to **5** that carries the *n*-pentyl residue. Atranorin (**3**) missing a mandatory free carboxylic acid group completely loses any mPGES-1inhibitory activity but is recognized to be the main active compound in a traditional lichen preparation, inhibiting COX-1 and COX-2 in a 45% range at 45 µM [28].

Here, we show for the first time that ingredients of *C. monachorum* also inhibit 5-LO and NF-κB and these properties might contribute to the overall anti-inflammatory activity of the CM extract. Among the 11 secondary metabolites identified, the diaromatic polyphenols (**3** - **5**) were revealed as the main bioactive chemical class of this extract. The order of their inhibitory activities in CM on both targets within eicosanoid biosynthesis is **5** > **4** >>**3**, indicating that the potencies against mPGES-1 and 5-LO run in parallel as potent dual inhibitors of these enzymes.

Interestingly, perlatolic acid (**5**), with the low IC_50_ value of 0.4 µM for both 5-LO and mPGES-1, is even more potent as compared to the reference inhibitors MK886 (of mPGES-1) and zileuton (of 5-LO) in the applied assays. Such high potency, together with the dual functionality against 5-LO and mPGES-1, qualifies **5** as valuable pharmacological agent. Note that under the assay conditions chosen, effects of the compounds on transcription and translation of 5-LO can be excluded due to the short incubation period (15 min). 

Whereas atranorin (**3**) was found to be inactive against 5-LO before [29], baeomycesic acid, a depside structurally closely related to atranorin and distinguished by a free carboxylic acid group, has been previously described as 5-LO inhibitor with IC_50_ = 8.3 µM [30], assuming this structural feature as crucial for activity. Furthermore, it was shown previously that the depsidone lobaric acid exhibits 5-LO as well as 12-LO inhibiting properties albeit with lower potencies (IC_50_ ≥ 28.5 µM; [4]). 

Inhibition of NF-κB transactivation activity by depsides described here is unprecedented. NF-κB inhibition has been reported only for some lichen constituents from other chemical classes, e.g. triterpenes [31], dibenzofurans [32], but to the best of our knowledge, this is the first report of depsides interacting with this pathway. Both, **4** and **5** exhibited a pronounced suppression of NF-κB activation in the low micromolar range, with imbricaric acid (**4**) being the most potent inhibitor (IC_50_ 2.0 µM) followed by perlatolic acid (**5**, IC_50_ 7.0 µM); **3** only showed a moderate activity with an IC_50_ of 20 µM. Taking into account that IKK2 inhibition can be ruled out as molecular mechanism for the observed NF-κB inhibition by **4** and **5**, the underlying mode of NF-κB suppression still needs to be elucidated. 

By conducting a pilot study, we here show (Figure 5) that one of the identified substances with anti-inflammatory activity in *in vitro* assays, perlatolic acid (**5**), inhibits the inflammatory recruitment of leukocytes in a peritonitis model *in vivo*. This observed inhibition of leukocyte recruitment compares well with the reported inhibition of recruitment by other inhibitors of the NF-κB- [33] and 5-LO- signaling pathways [34] and with the suggestion that mPGES-1 might be a likely candidate for a role as PCAM-1 independent modulator of leukocyte recruitment [35]. More detailed future analysis of the bioactive depsides in diverse animal models of inflammation e.g. peritonitis addressing various relevant biomarkers including cytokines, chemokines and lipid mediators including PGs and LTs are planned to fully assess the *in vivo* anti-inflammatory potential.

Correlation of the contents of these most abundant constituents with 15.22% of **4** and 9.10% of **5** in CM underpins the potent activities of the extract, which in this study could to be streamlined to these two multi-targeting depsides. Intriguingly, they bundle the inhibitory *in vitro* potential of three prominent targets involved in inflammatory processes and, thus, warrant further preclinical analysis as multi-target anti-inflammatory leads.

## Supporting Information

Information S1 Data and site of collection, voucher number, amount of dried thalli and yield of crude extract of 17 identified lichen species (Table S1.) and Isolation of pure compounds from *C. monachorum*.
