# Supplementary material for: Imbricaric Acid and Perlatolic Acid: Multi-Targeting Anti-Inflammatory Depsides from Cetrelia monachorum
Source: PLoS One. 2013 Oct 9;8(10):e76929. doi: 10.1371/journal.pone.0076929 (PMC3793931; doi:10.1371/journal.pone.0076929)
Supplement: Information S1 [file pone.0076929.s001.docx]

**Supporting Information**

**Imbricaric Acid and Perlatolic Acid: Multi-targeting Anti-Inflammatory Depsides from *Cetrelia monachorum***

Sarah K. Oettl^a^, Jana Gerstmeier^b^, Shafaat Y. Khan^c^, Katja Wiechmann^b^, Julia Bauer^d^, Atanas G. Atanasov^e^, Clemens Malainer^e^, Ezzat M. Awad^c^, Pavel Uhrin^c^, Elke H. Heiss^e^, Birgit Waltenberger^a^, Daniel Remias^a^, Johannes M. Breuss^c^, Joel Boustie^f^, Verena M. Dirsch^e^, Hermann Stuppner^a^, Oliver Werz^b,*^, Judith M. Rollinger^a,*^

**Affiliation**

^a^ Institute of Pharmacy/Pharmacognosy, Center for Molecular Biosciences Innsbruck, Leopold-Franzens University of Innsbruck, Innsbruck, Austria

^b^ Chair of Pharmaceutical/Medicinal Chemistry, Institute of Pharmacy, Friedrich-Schiller-University of Jena, Jena, Germany

^c^ Institute of Vascular Biology and Thrombosis Research, Center for Biomolecular Medicine and Pharmacology, Medical University of Vienna, Vienna, Austria

^d^ Department of Pharmaceutical Analytics, Pharmaceutical Institute, University Tuebingen, Tuebingen, Germany

^e^ Department of Pharmacognosy, University of Vienna, Vienna, Austria

^f^ Institute of Chemical Sciences of Rennes, UMR 6226, Team PNSCM, University of Rennes 1, Rennes, France

**^*^** Corresponding authors:

Phone: +43 51250758407; Fax: +43 51250758499 ; E-mail: [Judith.Rollinger@uibk.ac.at](mailto:Judith.Rollinger@uibk.ac.at)

Phone: +49 3641949801; Fax: +49 3641949801; E-mail: [Oliver.Werz@uni-jena.de](mailto:Oliver.Werz@uni-jena.de)

Table of contents:

**Table S1.** Data and site of collection, voucher number, amount of dried thalli and yield of crude extract of 17 identified lichen species.

**Isolation of pure compounds from *C. monachorum***

Table S1. Data and site of collection, voucher number, amount of dried thalli and yield of crude extract of identified lichen species.

| Abbr. | Lichen species | Date of collection | Locality | GPS data | Voucher number | Amount of dried thalli [g] | Yield of crude extract [mg] |
| --- | --- | --- | --- | --- | --- | --- | --- |
| **CN** | *Cetraria nivalis* (L.) Ach. | 08/2011 | Ötztal, A | N 46° 52.28' | JR-20110811-A23 | 12.66 | 818.95 |
|  |  |  |  | E 11° 0.21' |  |  |  |
|  |  |  |  | H 2115 m |  |  |  |
| **CP** | *Cetraria pinastri* (Scop.) Gray | 10/2010 | Halltal, A | N 47° 20.00' | JR-20101015-A8 | 1.90 | 65.81 |
|  |  |  |  | E 11° 28.04' |  |  |  |
|  |  |  |  | H 1603 m |  |  |  |
| **CM** | *Cetrelia monachorum* (Zahlbr.) W.L. Culb. & C.F.Culb. | 07/2011 | Almtal, A | N 47° 44.3’ | JR-20110709-A18 | 13.00 | 1683.72 |
|  |  |  |  | E 13° 56.82’ |  |  |  |
|  |  |  |  | H 602 m |  |  |  |
| **CC** | *Cladonia carneola* (Fr.) Fr. | 08/2011 | Ötztal, A | N 46° 52.22' | JR-20110811-A20 | 10.22 | 790.06 |
|  |  |  |  | E 11° 01.83' |  |  |  |
|  |  |  |  | H 1944 m |  |  |  |
| **LI** | *Lepraria incana* (L.) Ach. | 10/2010 | Halltal, A | N 47° 19.63' | JR-20101015-A2 | 2.52 | 59.30 |
|  |  |  |  | E 11° 28.86' |  |  |  |
|  |  |  |  | H 1488 m |  |  |  |
| **LL** | *Lobaria linita* (Ach.) Rabenh. | 08/2011 | Ötztal, A | N 46° 52.28' | JR-20110811-A21 | 22.71 | 2834.09 |
|  |  |  |  | E 11° 01.96' |  |  |  |
|  |  |  |  | H 1985 m |  |  |  |
| **LP** | *Lobaria pulmonaria* (L.) Hoffm. | 10/2010 | Halltal, A | N 47° 19.88' | JR-20101015-A11 | 7.70 | 188.05 |
|  |  |  |  | E 11° 28.69' |  |  |  |
|  |  |  |  | H 1515 m |  |  |  |
| **NR** | *Nephroma resupinatum* (L.) Ach. | 10/2010 | Halltal, A | N 47° 19.71' | JR-20101015-A3 | 4.80 | 140.42 |
|  |  |  |  | E 11° 28.80' |  |  |  |
|  |  |  |  | H 1574 m |  |  |  |
| **PC** | *Parmelia caperata* (L.) Ach. | 12/2010 | Vinschgau, I | N 46° 37.87' | JR-20101015-A15 | 11.34 | 72.60 |
|  |  |  |  | E 10° 48.05' |  |  |  |
|  |  |  |  | H 981 m |  |  |  |
| **PH** | *Parmeliopsis hyperopta* (Ach.) Arnold | 10/2010 | Halltal, A | N 47° 19.88' | JR-20101015-A12 | 5.20 | 261.40 |
|  |  |  |  | E 11° 28.69' |  |  |  |
|  |  |  |  | H 1515 m |  |  |  |
| **PL** | *Peltigera leucophlebia* (Nyl.) Gyeln. | 10/2010 | Halltal, A | N 47° 20.40' | JR-20101015-A14 | 6.30 | 394.50 |
|  |  |  |  | E 11° 29.48' |  |  |  |
|  |  |  |  | H 1420 m |  |  |  |
| **PR** | *Peltigera rufescens* (Weiss) Humb. | 08/2011 | Ötztal, A | N 46° 52.23' | JR-20110811-A25 | 7.18 | 580.36 |
|  |  |  |  | E 11° 02.13' |  |  |  |
|  |  |  |  | H 2114 m |  |  |  |
| **PG** | *Platismatia glauca* (L.) W.L. Culb. & C.F. Culb. | 10/2010 | Halltal, A | N 47° 19.73' | JR-20101015-A5 | 6.50 | 382.73 |
|  |  |  |  | E 11° 28.58' |  |  |  |
|  |  |  |  | H 1630 m |  |  |  |
| **SA** | *Stereocaulon alpinum* Laurer | 08/2011 | Ötztal, A | N 46° 52.28' | JR-20110811-A22 | 9.40 | 699.25 |
|  |  |  |  | E 11° 0.21' |  |  |  |
|  |  |  |  | H 2115 m |  |  |  |
| **TV** | *Thamnolia vermicularis* (Sw.) Ach. ex Schaer. | 08/2010 | Ötztal, A | N 46° 49.17' | JR-20101015-A16 | 6.24 | 930.80 |
|  |  |  |  | E 10° 59.03' |  |  |  |
|  |  |  |  | H 2637 m |  |  |  |
| **UC** | *Umbilicaria cyindrica* (L.) Delise ex Duby | 08/2011 | Ötztal, A | N 46°52.23' | JR-20110811-A24 | 16.53 | 595.63 |
|  |  |  |  | E 11°02.13' |  |  |  |
|  |  |  |  | H 2114 m |  |  |  |
| **XE** | *Xanthoria elegans* (Link) Th. Fr. | 08/2011 | Ötztal, A | N 46°51.83' | JR-20110811-A27 | 6.70 | 642.08 |
|  |  |  |  | E 11°01.25' |  |  |  |
|  |  |  |  | H 1948 m |  |  |  |

**Isolation of pure compounds from *C. monachorum***

For phytochemical investigation of *C. monachorum* 13 g dried and ground thalli were extracted at room temperature with EtOH 96% using an ultrasonic bath (1 x 130 mL, 7 x 80 mL, 1 h each). Upon evaporation to dryness the crude extract (CM) yielded 1.68 g. 1.20 g of CM were fractionated by flash silica gel (Merck silica gel 60, 0.040 – 0.063 mm, 119 g) column chromatography (CC; 3.2 × 39 cm) using a step gradient of petroleum ether-CH_2_Cl_2_-acetone-MeOH (petroleum ether 100 mL; petroleum ether/CH_2_Cl_2_ 80:20; 65:35; 50:50; 35:65; 20:80 v/v 200 mL each; CH_2_Cl_2_ 200 mL CH_2_Cl_2_/acetone 99:1; 98:2; 97:3; 95:5; 90:10; 80:20; 70:30; 60:40; 50:50; 40:60; 30:70; 20:80; 10:90 v/v 100 mL each; acetone 900 mL; acetone/MeOH 90:10 v/v 900 mL) resulting in 15 fractions (A1 – A15) monitored by TLC (SiO_2_, *n*-hexane/diethyl ether/formic acid 5:4:1 v/v/v, vanillin-sulfuric acid, Merck TLC silica gel 60 F_254_). 200 mg of fraction A13 (elution volume 2180 – 2740 mL; 211.21 mg) were further separated by silica gel CC (3.2 × 44 cm) using a step gradient of CH_2_Cl_2_-ethyl acetate-acetone (CH_2_Cl_2_/ethyl acetate 40:60 v/v 1000 mL; CH_2_Cl_2_/ethyl acetate 30:70; 20:80 v/v 500 mL each; CH_2_Cl_2_/ethyl acetate 10:90 v/v; ethyl acetate; ethyl acetate/acetone 75:25; 50:50 v/v 300 mL each; acetone 1800 mL; acetone containing 0.05 % formic acid 800 mL; acetone containing 0.1% formic acid 400 mL) yielding in 10 fractions (B1 – B10). B7 (elution volume 4872 - 5616 mL; 23.36 mg) enriched with compound **4** and compound **5** was subjected to preparative HPLC (Dionex, UltiMate 3000, Dionex Softron GmbH) using a Synergi Polar-RP 80A column (10 × 250 mm; 4 µm particle size; Phenomenex, Torrance, CA) and a mobile phase of 0.05% aqueous formic acid (A) and methanol (gradient grade; Merck; B) applying a gradient of 0 - 5 min 95% B, to 15 min 100% B, 15 – 20 min 100% B, injecting volume was 20 µL, detection wavelength 235 nm, to give 3 fractions (C1 – C3). C2 yielded compound **4** (elution volume 19.4 – 20.3 mL; 11.29 mg; Rf: 0.65), and C3 yielded compound **5** (elution volume 21.0 – 23.2 mL; 6.01 mg; Rf: 0.71). 1.11 mg of compound **3** (Rf: 0.72) were obtained by purifying fraction A3 (elution volume 820 – 960 mL; 8.48 mg) by recrystallization from methanol. 16 mg of fraction A12 (elution volume 2040 – 2180 mL; 17.07 mg) were subjected to Sephadex^®^ LH20 (Pharmacia Biotech) CC (1.3 × 34 cm) with MeOH as mobile phase yielding 3 fractions (D1 – D3). D2 gave pure compound **6** (elution volume 61 – 81 mL; 2.19 mg; Rf: 0.74). A4 (elution volume 960 – 1120 mL; 62.70 mg) was separated by Sephadex^®^ LH20 CC (1.9 × 89 cm) with CH_2_Cl_2_/acetone (v/v; 85:15) as mobile phase affording 15.18 mg of compound **7** (elution volume 64 – 68 mL; Rf: 0.74). Similarly, the separation of fraction A8 (elution volume 1480 – 1680 mL; 7.19 mg) yielded 5.15 mg of compound **2** (elution volume 105 – 150 mL; Rf: 0.54). 36 mg of fraction A9 (elution volume 1680 – 1840 mL; 41.95 mg) were subjected to Sephadex^®^ LH20 CC (1.9 × 89 cm) with CH_2_Cl_2_/acetone (v/v; 85:15) as mobile phase to give 10 fractions (E1 – E10). E4 (elution volume 80.0 – 92.5 mL; 2.52 mg) was purified by Sephadex^®^ LH20 CC (1.2 × 42 cm) with MeOH as mobile phase affording compound **1** (elution volume 33.0 – 43.5 mL; 1.11 mg; Rf: 0.61). E9 (elution volume 180.0 – 277.5 mL; 8.89 mg) was further separated by Sephadex^®^ LH20 CC (1.8 × 51 cm) with CH_2_Cl_2_/acetone (v/v; 85:15) as mobile phase yielding 7 fractions (F1 – F7). F2 gave pure compound **9** (elution volume 94 – 114 mL; 1.21 mg; Rf: 0.56), F4 yielded 3.93 mg of compound **8** (elution volume 120 – 142 mL; Rf: 0.56). Fraction A11 (elution volume 1920 – 2040 mL; 41.28 mg) was subjected to silica gel CC (1.2 × 40 cm) with CH_2_Cl_2_/ethyl acetate (v/v; 80:20) as mobile phase to give 5 fractions (G1 – G5). G3 (elution volume 78 – 94 mL; 19.89 mg) was further separated by silica gel CC (1.2 × 44 cm) with CH_2_Cl_2_/ethyl acetate (v/v; 80:20) as mobile phase yielding 2.05 mg of compound **10** (elution volume 46 – 50 mL; Rf: 0.46) and 3.22 mg of compound **11** (elution volume 54 – 64 mL; Rf: 0.44).
